# Alterations in bacterial metabolites, cytokines, and mucosal integrity in the caecum of broilers caused by feed additives and host-related factors

**DOI:** 10.3389/fphys.2022.935870

**Published:** 2022-09-12

**Authors:** Yada Duangnumsawang, Jürgen Zentek, Wilfried Vahjen, Joan Tarradas, Farshad Goodarzi Boroojeni

**Affiliations:** ^1^ Institute of Animal Nutrition, Department of Veterinary Medicine, Freie Universität Berlin, Berlin, Germany; ^2^ Faculty of Veterinary Science, Prince of Songkla University, Hatyai, Songkhla, Thailand; ^3^ Animal nutrition, Institute of Agrifood Research and Technology IRTA, Constantí, Spain

**Keywords:** short chain fatty acids, goblet cells, mucosal immunity, cytokines, host-microbe interactions, commercial broilers

## Abstract

A total of 2,880 one-day-old male and female broiler chicks from two breeds, Ross308 and Cobb500 were randomly assigned to 72 pens. Broilers were offered three diets: a wheat-soybean diet without (CO), or with either a probiotic (probiotic; 2.4 x 10^9^ CFU/kg diet of *Bacillus subtilis* DSM32324 and DSM32325 and *B. amyloliquefaciens* DSM25840) or a phytobiotic (phytobiotic; grape extract with 165 ppm procyanidin and 585 ppm polyphenol) product. The trial was conducted with a 3 × 2 × 2 factorial arrangement of diet, breed and sex in a completely randomized design and consisted of 6 replicate-pens per treatment (40 birds per pen). At day 7, 21, and 35, one chicken per pen was slaughtered for caecal sampling to quantify bacterial metabolites (digesta) as well as evaluate mRNA abundance and histomorphology (tissue). Data were subjected to ANOVA using GLM procedure to evaluate age, diet, breed and sex and their interactions. Spearman’s correlation (r) was analyzed between metabolite concentration and mRNA abundance. Overall, the concentration of short chain fatty acids increased with age, while lactate decreased from day 7 to 21 (*p* < 0.05). The mRNA abundance of IL-2, IL-4, IL-6, IL-8, IL-10, IL-12, IL-17α, IL-18, IFN-γ and TGF-β2 increased with age but IL-1β and TNF-α increased in abundance from day 7 to 21 and then decreased (*p* < 0.05). Abundance of MUC2 and CLDN5 increased after day 21 (*p* < 0.05). Caecal crypt depth increased with age (*p* < 0.05). Acidic goblet cell (GC) number peaked at day 21 (*p* < 0.05), while mixed GC number was not affected by age. A few impacts of breed, diet and interactions on the investigated variables showed no meaningful biological pattern. Propionate positively correlated with all cytokines investigated (r = 0.150–0.548), except TNF-α. Lactate negatively correlated with pro-inflammatory cytokines like IL-1β (r = −0.324). Aging affected caecal histomorphology, bacterial activity and genes responsible for barrier integrity and inflammatory response. This effect could be attributed to the interaction between gut microbiota and immune system as well as the direct effect of metabolites on gut histomorphology and cytokine mRNA abundance.

## 1 Introduction

Probiotics and phytobiotics have been used as potential substitutes for antibiotic growth promoters, with the goal to improve animal health and performance. The health advantages of probiotics are suggested to be related to their ability to modify the gut microbiota and its metabolic activity, as well as their subsequent role in modulation of the immune system (Lee et al., 2015; Park et al., 2020). Probiotics from genus *Bacillus* spp. have been receiving a great interest lately, because of their spore-forming abilities, which give them a number of advantages in terms of viability and stability during feed processing and also in the gut (Goodarzi Boroojeni et al., 2016; Zentek and Goodarzi Boroojeni, 2020). Adding *Bacillus* spp. to broiler feed inhibit intestinal pathogens, modify the bacterial community and their metabolic activity, diminish gut inflammation, modify mucosal morphology, and finally improve growth performance (Song et al., 2014; Park et al., 2020). Phytobiotics are a diverse group of plant-based products (including essential oils, herbs, and fruit extracts) with demonstrated health effects on gut health through their antioxidant, anti-inflammatory, and antibacterial properties (Viveros et al., 2011). Plant polyphenols such as procyanidins improve gut immunity through modifying intestinal microbiota, reducing oxidative stress, and modulating the expression of cytokines in the gut (Gessner et al., 2017). Procyanidins, the primary polyphenols in grape extract, can be catabolized by the intestinal microbiota into phenolic acids and other metabolites that help reducing oxidative stress and inflammation in the broilers gut (Chamorro et al., 2019; Cao et al., 2020). Grape polyphenols also found to increase short chain fatty acids (SCFA), and regulate the immune response and gut barrier integrity in broilers (Yang J. Y. et al., 2017; Cao et al., 2020).

The interactions of gut microbiota with their host affect immune responses, gut morphology and integrity (Apajalahti and Vienola, 2016). Adaptations in the intestinal microbial population occur concurrently with broiler growth. In newly hatched chicks, the gut bacterial community was already present but could only be characterized by limited bacterial diversity. However, the bacterial community composition, diversity and richness evolved over time (Glendinning et al., 2019). There are also some evidences showing that broiler’s intestinal microbiota can be affected by host genotype (Emami et al., 2022) and sex (Lumpkins et al., 2008). Despite the fact that commercial broilers are co-selected for performance and immunocompetence, their genetic make-up still affects their immune response to certain challenges (Cheema et al., 2003; Mayahi et al., 2016). For instance, different immunological developments and inflammatory responses (e.g. expression of pro-inflammatory cytokines) in Ross308 and Cobb500 have been attributed to their differences in gut microbial composition and activity (Hong et al., 2012; Richards et al., 2019). On the other hand, different characteristics in the gut morphology, such as villus height and crypt depth (Mabelebele et al., 2017), as well as distinct immunological traits and response to pathological challenges (Hong et al., 2012) contribute to a breed-specific bacterial community. Therefore, bacterial community and immunological status of different commercial breeds that are reared under the same environmental and nutritional conditions, may still differ. Generally, male broilers are known to have a higher growth rate and final body weight than females. This difference on growth rate between sexes may have an effect on composition of gut bacteria (Lumpkins et al., 2008). Since host-related factors can modulate the composition of gut microbes and gut immune responses, the environmental factors such as dietary treatment may interact with them and boost or discount their impacts. Hence, this study was conducted to evaluate the effect of feed additives (probiotics and phytobiotics), host-related parameters (age, breed, and sex) and their interactions on mucosal morphology, goblet cell number, bacterial metabolites, and mRNA abundance of cytokines, Mucin 2 (MUC2) and Claudin 5 (CLDN5) in the caecum of broilers. Additionally, the relationship between bacterial metabolites and cytokine responses in the gut was investigated, which may describe the interactions between gut microbiota and immune system.

## 2 Materials and methods

### 2.1 Animals and experimental diets

A total of 2,880 one-day-old male and female broiler chicks consisting of 1,440 Ross308^®^ (RS) and 1,440 Cobb500^®^ (CB) were randomly allocated into 72 pens (2.25 m^2^) with a softwood shaving floor. Three experimental diets including a standard wheat-soybean based diet without (CO) or with supplementation of either a probiotic (PO) or a phytobiotic (PY) product were produced and randomly assigned to birds. The trial was conducted with a 3 × 2 × 2 factorial arrangement of diet, breed and sex in a completely randomized design and consisted of 6 replicate-pens per treatment and 40 birds per pen (24 replicate-pens per diet, 36 replicate-pens per sex and 36 replicate-pens per breed). The experiment lasted 37 days. The experimental diets (starter diets for day 0–7, grower diets for day 8–21 and finisher diets for day 22–37) were formulated (Table 1) to meet or exceed recommendations of FEDNA (2018). The diets were offered in crumble form for the starter period and in 3 mm pellets later on. The probiotic product (GalliPro EPB5, Chr. Hansen, Denmark) which consists of *Bacillus subtilis* DSM32324 and DSM32325 and *B. amyloliquefacens* DSM25840 was added into the PO diets at a dosage of 2.4 x 10^9^ CFU/kg diet. The phytobiotic product (NutriPhy® White Grape 100, Chr. Hansen, Denmark) was included into the PY diets making a final concentration of 165 ppm procyanidin and 585 ppm total polyphenol in the diets. The applied dosages were according to the manufacturer recommendation.

**TABLE 1:** Dietary ingredients and nutrient composition.

Ingredients (%)	Starter (0–7 days old)	Grower (8–21 days old)	Finisher (22–37 days old)
Wheat	52.8	61.2	62.0
Soybean meal (48 % CP)	39.4	30.5	15.9
Soybean oil	4.16	4.80	0.00
Animal Fat (5 SYSFEED) ^a^	-	-	4.01
Extruded soybean	-	-	15.00
Dicalcium phosphate	1.85	1.66	1.50
Calcium carbonate	0.53	0.48	0.44
Vitamin-mineral premix ^b^	0.40	0.40	0.40
Sodium chloride	0.37	0.37	0.35
dl-methionine	0.27	0.23	0.19
l-lysine HCl	0.16	0.19	0.15
l-threonine	0.05	0.05	0.04
Choline chloride	0.03	0.05	0.05
Antioxidant (Noxyfeed 56P) ^c^	0.02	0.02	0.02
Sodium bicarbonate	-	0.010	0.002
Calculated nutrients			
AME, kcal/kg	2900	3000	3100
Lysine, g/kg	14.2	12.1	10.8
Methionine + cysteine, g/kg	10.1	8.8	8.1
Threonine, g/kg	9.3	7.9	7.2
Calcium, g/kg	9.6	8.7	8.1
Total phosphorus, g/kg	6.9	6.3	6.0
Sodium, g/kg	1.6	1.6	1.6
Analyzed nutrients			
Dry matter, g/kg	892	894	901
Crude protein, g/kg	245	213	201
Ether extract, g/kg	57	63	84
Ash, g/kg	58	52	49

a Product of Sysfeed SLU (Granollers, Spain), containing 1.5% myristic acid (C14:0), 18% palmitic acid (C16:0), 2% palmitoleic acid (C16:1 n-7), 14% stearic acid (C18:0), 28% oleic acid (C18:1 n-9 cis), 12% linoleic acid (C18:2 n-6 cis) and 6% α-linolenic acid (C18:3 n-3 cis).

b One kg of feed contains: Vitamin A: 10,000 IU; Vitamin D3: 4 800 IU; Vitamin E: 45 mg; Vitamin K3: 3 mg; Vitamin B1: 3 mg; Vitamin B2: 9 mg; Vitamin B6: 4.5 mg: Vitamin B12: 40 μg; Folic acid: 1.8 mg; Biotin: 150 μg; Calcium pantothenate: 16.5 mg; Niacin: 65 mg; Mn (as MnSO4.H2O): 90 mg; Zn (as ZnO): 66 mg; I (as KI): 1.2 mg; Fe (as FeSO4.H2O): 54 mg; Cu (as CuSO4.5H20): 12 mg; Se (as NaSeO3): 0.18 mg; BHT: 25 mg; Calcium formiate, 5 mg; Silicic acid, dry and precipitated, 25 mg; Calcium stearate, 25 mg; Calcium carbonate to 4 g

c Product of Itpsa (Barcelona, Spain), containing 56% of antioxidant substances (butylated hydroxytoluene + propyl gallate), 14% of citric acid and 30% of sepiolite as carrier.

### 2.2 Sample collection

Six birds per pen were randomly selected at 7, 21 and 35 days of age and the one with the closest body weight to the averaged pen-weight was used for the intended analysis. The birds selected for the analysis were sacrificed to dissect the caecum. The digesta was collected from proximal part of the right caecum and subsequently were frozen in liquid nitrogen and stored at -80°C until further analysis to quantify metabolite concentration. The caecal tissue collected from distal part of the right caecum was used for histomorphological analyzes. Tissues were fixed in 4 % (vol:vol) phosphate-buffered formaldehyde immediately after slaughtering and then transferred to 70 % ethanol until further analysis. Distal part of the left caecal tissue was collected for measuring mRNA abundance related to epithelial barrier proteins and inflammatory markers. Then, the tissue was stored in RNAlater buffer at -80°C until further analysis.

### 2.3 Histomorphological analyzes

The tissue samples were dehydrated, cleared with xylene and embedded with paraffin. Serial of 3 µm sections were prepared, mounted on glass slides and stained with Alcian blue-periodic acid-Schiff (AB-PAS) following manufacture’s protocol (AB-8GX, Sigma; Schiff’s reagent, Merck, Darmstadt, Germany).

Ten crypts from each caecal sample were selected for histomorphological analysis. Crypt depth (CD) was defined as its invagination depth. The number of acidic (blue), neutral (pink), mixed (purple), and total goblet cells (GC) in each crypt was counted. The density of GC was calculated from the number of GC per crypt divided by 100 µm of CD. All measurements were performed with an Olympus light microscope (BX 43, Olympus, Germany), which was equipped with a digital camera (DP72, Olympus, Germany). Image analysis was performed by using cellSens Standard software (version 1.14, Olympus, Germany) and ImageJ software (Rasband, W.S. ImageJ, United States National Institutes of Health, Bethesda, Maryland, United States).

### 2.4 Metabolite analyzes

Analysis of SCFA was performed by gas chromatography on an Agilent 6890 gas chromatography system with flame ionization detector and autosampler (Agilent Technologies, Böblingen, Germany), using the method described by Goodarzi Boroojeni et al. (2014). d- and l-lactate were analyzed by high-performance liquid chromatography on an Agilent 1100 chromatograph equipped with a Phenomenex C18 (4.0 × 2.0 mm^2^) guard column followed by a Phenomenex Chirex 3126 (d)-penicillamine column (150 × 4.6 mm^2^) and a UV detector at 253 nm, using the method described by Goodarzi Boroojeni et al. (2014).

### 2.5 RNA isolation and real time-quantitative PCR

The total RNA of caecal tissue was extracted by using NucleoSpin® RNA Plus kit and NucleoSpin® RNA clean-up (Macherey-Nagel GmbH & Co. KG, Düren, Germany). The mRNA quality and quantity were analyzed by a Bioanalyzer (Agilent 2100, Agilent, Waldbronn, Germany). Subsequently, reverse transcription of total RNA into cDNA in a final volume of 40 μl was executed using the Super Script III Reverse Transcriptase First-Strand cDNA Synthesis System (Invitrogen, Carlsbad, California). Primers used for the interleukin (IL)-1β, IL-2, IL-4, IL-6, IL-8, IL-10, IL-12, IL-17α, IL-18, tumor necrosis factor-α (TNF-α), interferon γ (IFN-γ), transforming growth factor-beta 2 (TGF-β2), MUC2 and CLDN5 are presented in Table 2. The RT-qPCR was conducted with a Stratagene MX3000p (Stratagene, Amsterdam, Netherlands). The reference genes β-actin, glycerinaldehyde-3-phosphate-dehydrogenase (GAPDH) and β2-microglobulin were used for normalization and times-fold abundance was determined based on mean cycle threshold values of the housekeeping genes using the relative abundance software tool REST^©^ (Pfaffl, 2002). The mRNA abundance was calculated as copy number per ng of total RNA. Then this value was divided by mean copy number of house-keeping genes to compare the abundance of targeted genes in different treatment groups.

**TABLE 2:** Primer sequences used for RT-PCR analysis.

Targets^a^	Sequences of primers (5′–3′)	A_T_ ^b^	References
IL-1β	GACATCTTCGACATCAACCAG CCGCTCATCACACACGACAT	60	Institute of Animal Nutrition, Freie Universität Berlin
IL-2	TCTGGGACCACTGTATGCTCT ACACCAGTGGGAAACAGTATCA	60	Hong et al. (2006)
IL-4	AACATGCGTCAGCTCCTGAAT TCTGCTAGGAACTTCTCCATTGAA	60	Avery et al. (2004)
IL-6	CTGCAGGACGAGATGTGCAA AGGTCTGAAAGGCGAACAGG	60	Institute of Animal Nutrition, Freie Universität Berlin
IL-8	GGCTTGCTAGGGGAAATGA AGCTGACTCTGACTAGGAAACTGT	60	Hong et al. (2006)
IL-10	GGAGGTTTCGGTGGAAGGAG GTTAAGCTGCCATTGAGCCG	60	Institute of Animal Nutrition, Freie Universität Berlin
IL-12	AGACTCCAATGGGCAAATGA CTCTTCGGCAAATGGACAGT	60	Hong et al. (2006)
IL-17α	AAGCGGTTGTGGTCCTCAT CTCCGATCCCTTATTCTCCTC	60	Hong et al. (2006)
IL-18	GGAATGCGATGCCTTTTG ATTTTCCCATGCTCTTTCTCA	60	Hong et al. (2006)
TNF-α	CTCGTTGGTGTGGGACGAC CGGCGGCGTATCGAAGTA	60	Institute of Animal Nutrition, Freie Universität Berlin
IFN-γ	CTCCCGATGAACGACTTGAG CTGAGACTGGCTCCTTTTCC	60	Sadeyen et al. (2004)
TGF-β2	TGCACTGCTATCTCCTGA ATTTTGTAAACTTCTTTGGCG	60	Sundaresan et al. (2008)
MUC2	TGGCTGTGTAACTGCACCAA GTGGGTTTAGGAGGTGGCTC	60	Institute of Animal Nutrition, Freie Universität Berlin
CLDN5	CATCACTTCTCCTTCGTCAGC GCACAAAGCTCTCCCAGGTC	60	Institute of Animal Nutrition, Freie Universität Berlin
β-actin	GAGAAATTGTGCGTGACATCA CCTGAACCTCTCATTGCCA	60	Li et al. (2005)
GAPDH	GGTGGTGCTAAGCGTGTTA CCCTCCACAATGCCAA	60	Li et al. (2005)
β2-microglobulin	AAGGAGCCGCAGGTCTAC CTTGCTCTTTGCCGTCATAC	60	Li et al. (2005)

a Three reference genes including β-actin, GAPDH and β2-microglobulin were used as house-keeping genes.

b A_T_, annealing temperature (°C)

IL, interleukin; TNF-α, tumor necrosis factor alpha; IFN-γ, interferon gamma; TGF-β2, transforming growth factor beta 2; CLDN5, Claudin 5; MUC2, Mucin 2; GAPDH, glycerinaldehyde-3-phosphate-dehydrogenase.

### 2.6 Statistical analyzes

Statistical analyzes were conducted using SPSS 26 (SPSS Inc. Chicago, IL, United States). Data were subjected to ANOVA using GLM procedure to evaluate the main factors including three ages (day 7, 21 and 35 of age), three dietary treatments (CO, PO and PY), two breeds (RS and CB), and two sexes (male and female) and their interactions. Means were separated by the Tukey least significant difference post hoc test at *p* < 0.05 statistical level. Means and pooled standard error of the mean (SEM) were reported for all variables measured. Replicate-pen was the experimental unit for all variables measured.

Spearman’s rank correlation coefficients (expressed as r) were used to assess associations between bacterial metabolites and mRNA abundance of the investigated genes, using the Spearman’s test in SPSS 26 and illustrated in GraphPad Prism 9.0.2 for Windows (GraphPad Software, San Diego, California United States). A *p*-value below 0.05 was considered as statistically significant.

## 3 Results

The results of histomorphological analysis in the caecum is shown in Table 3. Overall, the averaged CD in the caecum increased with age (*p* < 0.05) by 105 % from day 7 to 35. No effect of dietary treatment, breed and sex was observed on the caecal CD (*p* > 0.05). The majority of GC presented in the caecum was mixed GC (61.2 %–85.2 %) and the remaining was acidic. Neutral GC was not present in most of the samples and when present, their number was negligible. The number of acidic GC per crypt was increased by 39 % from day 7 to 21 of age and then decreased by 38 % at 35 days of age (*p* < 0.05). The number of mixed and total GC per crypt was not affected by age (*p* > 0.05), but numerically increased from 7 to 35 days of age. For the GC density (no. of cells/100 um CD), the mixed and total GC density decreased by 39.4 % and 34.3 % from day 7 to 21 of age, respectively (*p* < 0.05) and no further changes were found at day 35 of age (*p* > 0.05). The acidic GC density decreased by 20.0 % from day 7 to 21 and by 42.9 % from day 21 to 35 (*p* < 0.05). Breed effect was found for mixed and total GC density, with a greater density in RS by 11.3 % and 7.37 %, respectively compared with CB (*p* < 0.05). The number of mixed GC per crypt was also 13.5 % higher in RS than CB (*p* < 0.05). Dietary treatment and sex had no impact on GC number and density in the caecal crypt (*p* > 0.05). There was interaction between age and dietary treatment for mixed and total GC density (*p* = 0.032 and 0.049, Supplementary Table S3A,B) and between age, breed, sex and dietary treatment for mixed GC density (*p* = 0.038, Supplementary Table S3C). Broilers showed a greater mixed GC density at 7 days of age than those at 21 and 35 days of age regardless of dietary treatment (*p* > 0.05), except those 7 days old birds receiving the CO and PY diets were not different from 35 days old birds feeding the PY diet (*p* > 0.05). Similarly, the density of total GC in 7 days old broilers was also greater than in older birds, regardless of dietary treatment (*p* < 0.05), but 7 days old birds fed PY diet showed a similar total GC density to 21 days old birds receiving CO diet (*p* > 0.05). RS-male broilers at day 7 receiving PO diets showed highest mixed GC density and CB-male birds at day 35 receiving PY and PO diets displayed lowest mixed GC density CB (*p* < 0.05).

**TABLE 3:** The effect of age, dietary treatment, breed and sex on histomorphology in the caecum of broilers^a^.

Parameters^*^	Age (A)			Dietary treatment (T)			Breed (B)		Sex (S)		SEM	*p*-value			
	7	21	35	CO	PO	PY	RS	CB	M	F		A	T	B	S
***Caecal morphology***															
CD^b^	150^c^	257^b^	308^a^	263	257	251	254	260	261	253	5.5	<0.001	0.929	0.652	0.325
***Goblet cell number*** ***^c^***															
Acidic	5.2^b^	7.2^a^	4.5^b^	6.0	5.9	5.3	5.6	5.8	5.8	5.7	0.24	<0.001	0.477	0.835	0.643
Mixed	14.2	15.2	17.2	15.6	15.6	16.1	16.8^a^	14.8^b^	15.6	16.0	0.45	0.052	0.921	0.032	0.741
Total	19.4	22.4	21.6	21.6	21.5	21.4	22.4	20.6	21.4	21.6	0.51	0.157	0.975	0.072	0.627
***Goblet cell density*** ***^d^***															
Acidic	3.5^a^	2.8^b^	1.6^c^	2.5	2.6	2.3	2.4	2.5	2.4	2.5	0.11	<0.001	0.355	0.604	0.339
Mixed	9.9^a^	6.0^b^	6.0^b^	6.5	6.9	6.8	7.1^a^	6.3^b^	6.6	6.9	0.22	<0.001	0.400	0.009	0.448
Total	13.4^a^	8.8^b^	7.5^b^	9.0	9.5	9.0	9.5^a^	8.8^b^	9.0	9.4	0.27	<0.001	0.376	0.047	0.279

a The trial was conducted with a 3 × 2 × 2 factorial arrangement of diet, breed and sex in a completely randomized design and consisted of 6 replicate-pens per treatment and 40 birds per pen. Data were subjected to ANOVA using GLM procedure to evaluate age, diet, breed and sex and their interactions.

b Crypt depth are measured in µm.

c The average number of goblet cells per caceal crypt. Acidic represents the cells that are positive to Alcian blue dye. Mixed represents the cells that are positive to both Alcian blue and PAS dye. Total represents the sum of acidic and mixed goblet cells.

d The average number of goblet cells per 100 µm length of the crypt depth. Acidic represents the cells that are positive to Alcian blue dye. Mixed represents the cells that are positive to both Alcian blue and PAS dye. Total represents the sum of acidic and mixed goblet cells.

^a,b,c^Means with different superscripts in a row within the main factor differ significantly (*p <* 0.05).

^*^CO, control; PO, probiotic product; PY, phytobiotic product; RS, Ross; CB, Cobb; M, male; F, female.

The effect of age, breed, sex and dietary treatment on metabolite content in the caecum is shown in Table 4. During the whole period of the study, acetate represented approximately 71–80 % of total SCFA concentration, followed by n-butyrate and propionate which represented 14–15 % and 4–11 % of total SCFA concentration, respectively. Concentration of all SCFA increased with age (*p* < 0.05), with the most drastic change in propionate (3.72-fold from 7 to 35 days of age), followed by n- and i-valerate, n-and i-butyrate, and acetate (2.67-, 1.59-, 1.53-, 1.38- and 1.19-fold, respectively). However, concentration of propionate, n-butyrate, i-valerate and total BCFA was stable in the caecum from 7 to 21 days of age (*p* > 0.05). Acetate concentration increased from day 7 to 21 (*p* < 0.05) and stayed stable from day 21 to 35 (*p* > 0.05). Furthermore, i-butyrate concentration at 21 days of age was not different from 7 or 35 days of age (*p* > 0.05). Dietary treatment, breed and sex had no impact on SCFA concentration (*p* > 0.05). There was no interaction between age, breed, sex, and dietary treatment for SCFA concentrations in the caecum (*p* > 0.05, Supplementary Table S4A).

**TABLE 4:** The effect of age, dietary treatment, breed and sex on metabolite concentration (µmol/g of fresh sample) in the caecum^a^.

Parameters^*^	Age (A)			Dietary treatment (T)			Breed (B)		Sex (S)		SEM	*p*-value			
	7	21	35	CO	PO	PY	RS	CB	M	F		A	T	B	S
***Short chain fatty acids***															
Acetate	57.67^b^	66.39^a^	68.90^a^	66.21	65.14	63.82	65.99	64.15	65.04	65.07	1.423	0.009	0.776	0.605	0.886
Propionate	2.95^b^	5.71^b^	10.98^a^	6.64	8.32	5.96	6.59	7.30	6.47	7.42	0.666	<0.001	0.562	0.607	0.764
i-butyrate	0.64^b^	0.68 ^ab^	0.88^a^	0.68	0.84	0.72	0.81	0.69	0.72	0.77	0.038	0.008	0.265	0.152	0.517
n-butyrate	10.13^b^	12.20^b^	15.47^a^	13.02	13.26	12.37	12.43	13.32	12.64	13.12	0.464	<0.001	0.556	0.461	0.602
i-valerate	0.37^b^	0.33^b^	0.59^a^	0.40	0.50	0.43	0.45	0.43	0.46	0.42	0.024	<0.001	0.283	0.774	0.339
n-valerate	0.30^c^	0.64^b^	0.81^a^	0.64	0.63	0.57	0.60	0.63	0.61	0.62	0.026	<0.001	0.226	0.400	0.816
Total SCFA^b^	72.06^c^	85.96^b^	97.64^a^	87.59	88.68	83.86	86.85	86.51	85.94	87.41	1.943	<0.001	0.567	0.953	0.651
Total BCFA^c^	1.01^b^	1.01^b^	1.48^a^	1.07	1.34	1.14	1.25	1.12	1.18	1.18	0.055	<0.001	0.172	0.272	0.996
***Lactate***															
l-lactate	1.09	0.42	0.85	0.87	0.66	0.73	0.84	0.66	0.69	0.81	0.107	0.075	0.774	0.465	0.641
d-lactate	1.64^a^	0.46^b^	0.56^b^	0.69	0.83	0.89	0.68	0.94	0.82	0.79	0.135	<0.001	0.857	0.121	0.701
Total lactate	2.68^a^	0.86^b^	1.41 ^ab^	1.53	1.49	1.61	1.50	1.59	1.48	1.60	0.223	0.006	0.995	0.600	0.959
d- to l-lactate ratio	1.49	1.30	0.95	0.87	1.71	1.01	0.92	1.50	1.04	1.33	0.204	0.550	0.334	0.223	0.556

a The trial was conducted with a 3 × 2 × 2 factorial arrangement of diet, breed and sex in a completely randomized design and consisted of 6 replicate-pens per treatment and 40 birds per pen. Data were subjected to ANOVA using GLM procedure to evaluate age, diet, breed and sex and their interactions.

b Total short chain fatty acid is the sum of acetate, propionate, i-butyrate, n-butyrate, i-valerate and n-valerate concentration.

c Total branched chain fatty acid is the sum of i-butyrate and i-valerate concentration.

^a,b,c^Means with different superscripts in a row within the main factor differ significantly (*p <* 0.05).

^*^CO, control; PO, probiotic product; PY, phytobiotic product; RS, Ross; CB, Cobb; M, male; F, female.

Concentration of caecal d- and total lactate decreased from day 7 to 21 of age by 72.0 and 67.9 %, respectively (*p* < 0.05). Concentration of caecal d-lactate was identical for 21 and 35 days old broilers, while concentration of total lactate in the caecum of 35 days old broilers was similar to 7 and 21 days old broilers (*p* > 0.05). l-lactate and the ratio of d- to l-lactate were not different among age groups (*p* > 0.05). Dietary treatment, breed and sex as well as the interactions between the main factors showed no effect on lactate concentrations in the caecum (*p* > 0.05).

**FIGURE 1:**
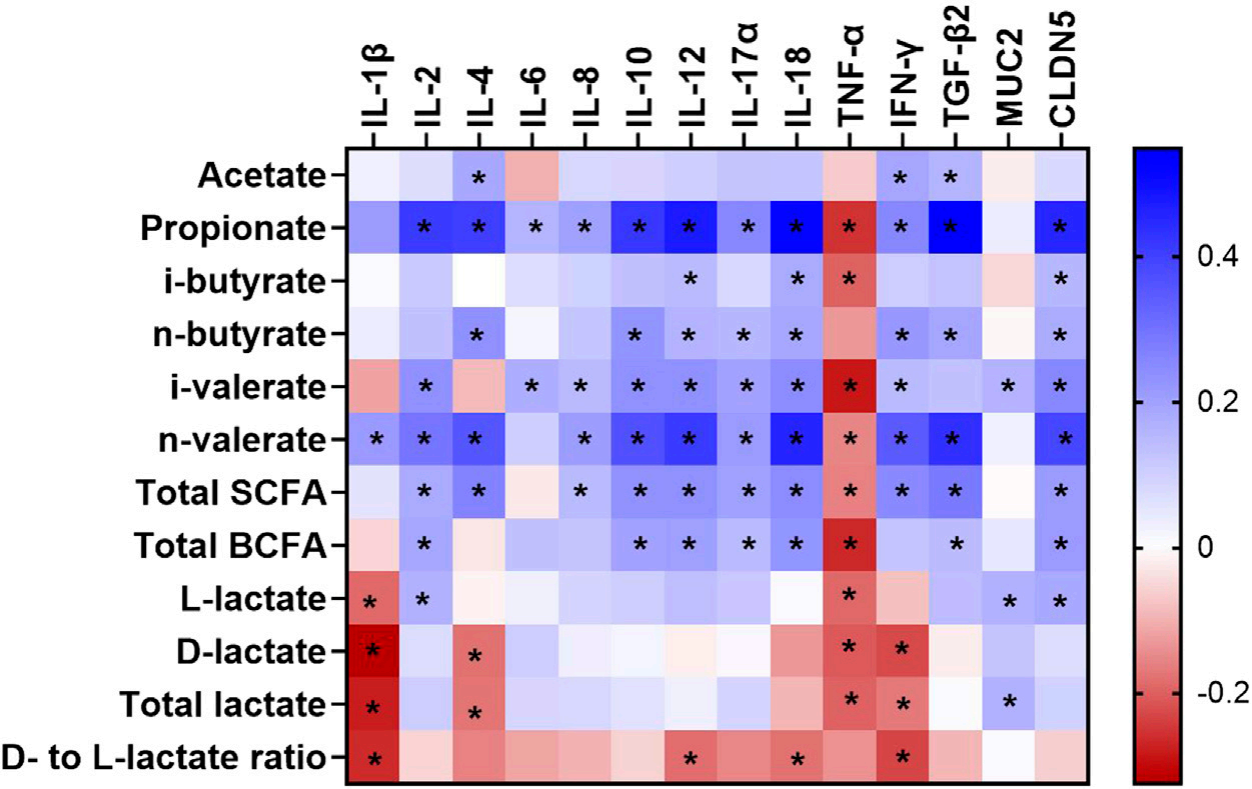
A heatmap showing the Spearman’s correlation coefficient between metabolites and mRNA abundance in the caecum of broilers between day 7 and 35 of age. The colors represent the correlation, with blue being more positive and red being more negative. Significance is given as * (*p* < 0.05). SCFA, short chain fatty acid; BCFA, branched chain fatty acid; IL, interleukin; TNF-α, tumor necrosis factor alpha; IFN-γ, interferon gamma; TGF-β2, transforming growth factor beta 2; CLDN5, Claudin 5; MUC2, Mucin 2.

The impact of age, dietary treatment, sex and breed on mRNA abundance related to epithelial barrier function and inflammatory markers of the caecum is shown in Table 5. The mRNA abundance of all cytokines (IL-1β, IL-2, IL-4, IL-6, IL-8, IL-10, IL-12, IL-17α and IL-18 as well as IFN-γ and TGF-β2) as well as MUC2 and CLDN5 increased from day 7 to 35 of age, except for TNF-α (*p* < 0.05). However, the abundance of several mRNA including IL-2, IL-6, IL-8, IL-10, IL-17α and CLDN5 was stable from day 7 to 21 of age (*p* > 0.05), while MUC2 decreased by 1.46-fold during this time (*p* < 0.05). Although most of mRNA abundance were increased from day 21 to 35 of age, the abundance of IL-1β and TNF-α were decreased during this period (*p* < 0.05). Dietary treatment only affected IL-10 abundance, with higher level in birds receiving PY diet compared with those fed CO diet (*p* < 0.05). Abundance of IL-1β, IL-6, TNF-α and CLDN5 was higher in CB than RS (*p* < 0.05). Sex had no impact on the mRNA abundance measured (*p* > 0.05). The only significant interaction was between age, treatment and sex on MUC2 abundance (*p* < 0.05, Supplementary Table S5A,B). At day 21, female birds fed CO diets, as well as male and female birds fed PY diets, expressed less MUC2 abundance level than birds aged 7 days (male birds fed PO diets and female birds fed PY diets) and 35 days (irrespective of diet or sex) (*p* < 0.05). In contrast, the abundance of MUC2 of male birds fed CO diet as well as male and female birds fed PO diet at day 21 of age were not different from the other age groups regardless of dietary treatment or sex (*p* > 0.05). The interaction between age, breed and sex was significant for IL-2 (*p* < 0.05). The highest abundance level of IL-2 was observed in 35 days old birds compared with 7 and 21 days old broilers. At day 21, female RS and male CB broilers showed a lesser IL-2 abundance than 7 days old broilers, regardless of their breed or sex (Supplementary Table S5C). The correlations between metabolite concentration and mRNA abundance were also analyzed as shown in Figure 1 (also Supplementary Table S6). The SCFA concentrations in the caecum, predominantly propionate, showed weak to moderate positive correlations (r = 0.150 to r = 0.548) with all the mRNA measured (*p* < 0.05), except for TNF-α, while its correlations with IL-18 (r = 0.518) and TGF-β2 (r = 0.548) were pronounced. The mRNA abundance of TNF-α showed negative correlations (r = −0.154 to r = −0.285, *p* < 0.05) with all the metabolites measured (*p* < 0.05) except for acetate, n-butyrate and d- to l-lactate ratio. Acetate as the predominant SCFA in the caecum, showed weak but positive correlations (*p* < 0.05) with IL-4, IFN-γ and TGF-β2 (r = 0.185, 0.191 and 0.157, respectively). There were only a few significant correlations between lactate and the investigated mRNA (*p* < 0.05) which were mainly weak (r = 0.183 to r = -0.324, respectively). d- and total lactate concentration was negatively correlated (*p* < 0.05) with IL-1β (r = −0.324 and r = −0.279, respectively), IL-4 (r = −0.179 and r = −0.175, respectively), TNF-α (r = −0.209 and r = −0.198, respectively) and IFN-γ (r = −0.227 and r = −0.170, respectively), while total lactate was positively correlated (r = 0.166) with MUC2 (*p* < 0.05). l-lactate concentration was negatively correlated (*p* < 0.05) with IL-1β (r = -0.189) and TNF-α (r = -0.190), but it showed a positive correlation (*p* < 0.05) with IL-2 (r = 0.166), MUC2 (r = 0.167) and CLDN5 (r = 0.183).

**TABLE 5:** The impact of age, dietary treatment, sex and breed on expression of the genes (log_10_ copy number per ng of RNA^a^) related to epithelial barrier function and inflammatory markers of the caecum*

Parameters**	Age (A)			Dietary treatment (T)			Breed (B)		Sex (S)		SEM	*p*-value			
	7	21	35	CO	PO	PY	RS	CB	M	F		A	T	B	S
***Cytokines***															
IL-1β	−3.30^c^	−2.55^a^	−2.99^b^	−2.98	−2.92	-2.94	−3.00^b^	−2.90^a^	−2.97	−2.92	0.029	<0.001	0.360	0.020	0.293
IL-2	−5.00^b^	−5.10^b^	−4.06^a^	−4.71	−4.76	-4.68	−4.70	−4.74	−4.70	−4.74	0.037	<0.001	0.317	0.314	0.269
IL-4	−4.35^b^	−3.50^a^	−3.54^a^	−3.81	−3.83	-3.76	−3.82	−3.78	−3.81	−3.79	0.029	<0.001	0.209	0.255	0.847
IL-6	−5.29^b^	−5.36^b^	−4.95^a^	−5.24	−5.09	-5.27	−5.29^b^	−5.11^a^	−5.18	−5.22	0.040	<0.001	0.148	0.028	0.565
IL-8	−2.66^b^	−2.65^b^	−2.23^a^	−2.53	−2.46	-2.54	−2.53	−2.50	−2.51	−2.51	0.027	<0.001	0.344	0.569	0.897
IL-10	−6.23^b^	−6.24^b^	−4.87^a^	−5.87^b^	−5.74^ab^	-5.71^a^	−5.80	−5.74	−5.80	−5.74	0.050	<0.001	0.004	0.163	0.429
IL-12	−4.60^c^	−4.45^b^	−3.39^a^	−4.16	−4.14	-4.14	−4.15	−4.14	−4.16	−4.13	0.040	<0.001	0.774	0.985	0.595
IL-17α	−3.81^b^	−3.90^b^	−3.07^a^	−3.63	−3.52	-3.63	−3.57	−3.62	−3.65	−3.54	0.045	<0.001	0.398	0.536	0.158
IL-18	−3.09^c^	−2.75^b^	−2.24^a^	−2.74	−2.67	-2.67	−2.70	−2.68	−2.69	−2.70	0.028	<0.001	0.072	0.645	0.691
TNF-α	−3.22^b^	−2.82^a^	−3.41^c^	−3.19	−3.14	-3.12	−3.21^b^	−3.09^a^	−3.15	−3.15	0.022	<0.001	0.098	<0.001	0.906
IFN-γ	−3.21^b^	−2.61^a^	−2.50^a^	−2.81	−2.77	-2.74	−2.75	−2.80	−2.76	−2.78	0.032	<0.001	0.505	0.215	0.550
TGF-β2	−1.43^c^	−0.96^b^	−0.73^a^	−1.08	−1.04	-0.99	−1.06	−1.02	−1.03	−1.05	0.025	<0.001	0.102	0.302	0.514
***Gut barrier related proteins***															
MUC2	−0.83^b^	−1.21^c^	−0.70^a^	−0.96	−0.88	−0.93	−0.93	−0.91	−0.90	−0.94	0.027	<0.001	0.311	0.712	0.364
CLDN5	−2.19^b^	−2.17^b^	−1.48^a^	−1.97	−1.95	−1.91	−1.98^b^	−1.91^a^	−1.94	−1.95	0.026	<0.001	0.220	0.017	0.703

a Log_10_ copy number per ng of RNA was calculated by dividing the amount of mRNA (copy number per ng of RNA) of targeted genes with the amount of mRNA of house keeper genes and then the obtained value was transformed to log_10_ scale.

^a,b,c^Means with different superscripts in a row within the main factor differ significantly (*p <* 0.05).

*Results are reported as means of 6 replicate-pens. The trial was conducted with a 3 × 2 × 2 factorial arrangement of diet, breed and sex in a completely randomized design and consisted of 40 birds per pen. One bird per pen for each group were subjected to ANOVA using GLM procedure to evaluate age, diet, breed and sex and their interactions.

**CO, control; PO, probiotic product; PY, phytobiotic product; RS, Ross; CB, Cobb; M, male; F, female; IL, interleukin; TNF-α, tumor necrosis factor alpha; IFN-γ, interferon gamma; TGF-β2,transforming growth factor beta 2; CLDN5, Claudin 5; MUC2, Mucin 2

## 4 Discussion

Chicken caecum is inhabited by complex microbial community. These organisms are known to produce metabolites modulating morphological structure along the gut (Shakouri et al., 2009) and interacting with gut immunity (Willson et al., 2018). Host-related factors including broiler breed, age and sex have been reported to affect intestinal microbiota and immune function (Torok et al., 2013). In this study, the impact of genetic background (breed and sex) was barely observed on caecal bacterial metabolites, histomorphology, integrity and immunological traits. There were no differences between males and females for all the variables measured in the current study. RS showed higher mixed and total GC density as well as higher number of mixed GC per crypt, compared with CB. Abundance of IL-1β, IL-6 and TNF-α (genes related to pro-inflammatory responses) as well as CLDN5 (one of the barrier-forming claudins) was increased in CB compared with RS. These few observed differences in intestinal phenotypes, mainly between breeds, did not show any meaningful biological pattern. The observed similarities between breeds and sexes might be attributed to the optimum rearing condition in the present study and absence of harmful stimuli, causing stress for bacterial population, stimulating the epithelial barrier function and triggering certain gut immune responses in order to protect the gut from additional injury (Mabelebele et al., 2017; Paraskeuas and Mountzouris, 2019; Wang et al., 2021).

Gut microbiota could directly or indirectly (via metabolites) interact with intestinal epithelium and modulate immune responses (Broom and Kogut, 2018). Beneficial impacts of probiotics and phytobiotics on poultry health and performance have been shown to be mainly through gut microbiota by supporting proliferation and metabolic activity of beneficial bacteria and decreasing the number and metabolic activity of those having harmful or pathogenic characteristics (Heak et al., 2018). In this study, probiotic and phytobiotic supplementation did not show any impact on caecal morphology, bacterial metabolic activity, and mRNA abundance, except for IL-10 which was increased in the caecum of birds receiving the phytobiotic product compared with birds in the control group. The cytokine IL-10 plays an essential role in anti-inflammatory response which regulates mucosal immune function (Lu et al., 2014). Grape extract has been reported to modulate cytokine expression through suppressing pro-inflammatory cytokines in the gut (e.g. IL-1β) of broilers (Cao et al., 2020) and increasing anti-inflammatory cytokines including IL-10 and TGF-β1 in human Caco-2 colon cells (Nallathambi et al., 2020). In several broiler studies, it has been shown that *B. subtilis* stimulated the effector and regulatory T cells and increased their cytokine production including IL-1β, IL-12, IFN-γ and IL-10 in the small intestine (Rajput et al., 2013; Lee et al., 2015) and caecum (Bilal et al., 2021). However, adding *B. subtilis* to broiler diets in the present study had no impact on mRNA abundance of all the investigated cytokines and intestinal barrier proteins as well as metabolic activity of bacteria in the caecum. In accordance with the present study, adding *B. subtilis* (Choi et al., 2021; Erinle et al., 2022) and grape seed extract (Cao et al., 2020) to broiler diets had almost no impact on caecal microbiota and their metabolic activity. Other studies, however, have shown the effect of *B. subtilis* on increasing bacterial SCFA concentration in the ileum (Aljumaah et al., 2020) and jejunum (Kan et al., 2021) of broilers. Grape extract was also found to affect bacterial metabolites in the gut of broilers through modulating phenolic metabolism of bacteria (Chamorro et al., 2019). The discrepancy in findings of different studies could be driven by qualitative differences in the extracts used, environmental factors like housing circumstances and diet composition, as well as host-related factors like age, breed, and sex, which differed between trials and could have impacted gut microbiota development (Kers et al., 2018).

In the current study, the observed alterations in the caecum’s morphology, mRNA abundance, and bacterial metabolites were mainly age-related. During co-development of the host and gut microbiota, products of bacterial metabolic activity, like SCFA and lactate, could be the main factors triggering the interaction between the host and gut microbiota (Yang L. et al., 2017). In the present study, acetate, propionate, and butyrate concentration increased during 35 days of age, while lactate concentration decreased from day 7 to 21 of age and remained stable afterward. The opposite age-related direction of SCFA (increase) and lactate (decrease) concentrations in the caecum was also previously reported and suggested to be due to a direct stimulation of lactate-utilizing bacteria or indirect action of bacterial groups playing role in metabolic cross-feeding of fermentation products (Meimandipour et al., 2011). Reduction of lactate concentration in the caecum during the first two weeks of age was attributed to replacement of lactic acid bacteria, especially *Lactobacillus*, with other dominant bacterial groups, mainly Clostridiaceae (Ranjitkar et al., 2016). The Clostridiaceae are well-known for conversion of complex polysaccharides to SCFA (Eeckhaut et al., 2011). Most of complex polysaccharides are indigestible in the small intestine of broilers thus, are available as substrates for microorganism in the hind gut. Basically, as chickens become older, the quality of their diets reduces and the concentration of indigestible polysaccharides in their diets increases. Dominance of Clostridiaceae and their increasing access to complex polysaccharides in the caecum can be one of the reasons for increasing SCFA concentration in the caecum of broilers. Furthermore, some bacteria in the Clostridiaceae group, like *Clostridium, Faecalibacterium*, and *Ruminococcus* spp. can utilize lactate as a substrate for butyrate production (Eeckhaut et al., 2011). Thus, the age-related shift from lactic acid bacteria to Clostridiaceae, may increase SCFA and butyrate and reduce lactate concentration, as also have been seen in the present study. Furthermore, it was suggested that the microbial community may exert different metabolic pathways at different ages depending on luminal state, microbial makeup, and host immune response (Wu et al., 2021). In the current study, age had a greater impact on propionate than other SCFA. Propionate concentration increased by 272 % from day 7 to 35 of age, whereas acetate and butyrate increased only by 19 % and 53 %, respectively. A pronounced increase in propionate over other SCFA was also reported in broilers during 3–6 weeks of age which was suggested to be due to alterations in bacterial composition that increased propionate producers and/or decreased lactobacilli (Meimandipour et al., 2010; Kim et al., 2020; Liao et al., 2020). Several studies have shown that as chickens age, the caecal microbial community becomes more diversified, with a greater number of distinct species (Lu et al., 2003; Oakley et al., 2014; Oakley and Kogut, 2016). The constant (age-related) alterations in metabolites concentration could also reflect that the caecal microbial community of broilers in the present study had not reached its mature and steady state even at 35 days of age. This speculation is in line with prior studies showing (by sequencing 16S rRNA) shifts in the caecal bacterial community (Lu et al., 2003; Oakley and Kogut, 2016) and increases in SCFA concentration (Liao et al., 2020) between 3 and 7 weeks of age. Other studies have also claimed that increasing SCFA concentrations in the caecum of older broilers (Liao et al., 2020) and laying hens (Sun et al., 2021) may reflect maturation process of the microbial community by age. However, there are studies demonstrating a “mature” stage of gut microbiota as early as 3 weeks of age, by comparing phylogenetic diversity in the caecum of 3 weeks old broilers with older (up to 6 weeks of age) birds (Kers et al., 2020) or by a regression model, using the microbiota maturation index for fecal microbiota (Gao et al., 2017). The inconsistency in outcome (the mature stage of gut microbiota) of the studies could be attributed to differences in analytical methodology as well as host genetics, diet, and environmental conditions, which could have affected microbial composition (Pedroso et al., 2005; Richards et al., 2019; Hou et al., 2020).

Intestinal morphology can be considered as a direct measure of intestinal health, as the mucosal epithelium regenerates to replace injured cells and constantly reshapes the mucosal structure in the gut. In the present study, morphology of the caecal crypt was changed with age; CD in the caecum increased by more than 100 % from day 7 to 35 of age. Alterations in the luminal environment by bacterial metabolites may affect villus or crypt structure and mucin production which are important defense structures in the gut (Iacob et al., 2019). As a source of energy, butyrate plays a vital role in promoting intestinal development and maintaining the integrity of the intestinal epithelial cells (Zou et al., 2019). Acetate has been shown to alter intestinal cell apoptosis and mucus production (Liu et al., 2017). Propionate is also a potent fatty acid that modulate intestinal cell activity including differentiation and apoptosis (Hosseini et al., 2011). The concentration of bacterial metabolites such as SCFA is usually higher in the caecum than other areas of the gastrointestinal tract, and their impact on gut histomorphology development should also be highest in the caecum compared with other sections of the gut (Parada Venegas et al., 2019). Therefore, age-related increases in SCFA concentration found in this study may have impacted formation of epithelial cells in the crypt of growing broilers and among other factors, stimulated morphological changes in their caecum. Intestinal GC is the first line of defense for the mucosa. Mucins produced by GC can protect epithelial cells from infections as well as chemical and mechanical damages (Duangnumsawang et al., 2021). In this study, the number of total GC per crypt was not affected by age. Another study found an increased number of caecal GC of 49 days old broilers compared with 28 days old broilers (Jiang et al., 2009). In contrast, Thiam et al. (2021) found that total number of GC per crypt in the ileum and caecum of broilers tended to decrease from day 7 to 21 of age. The changes in GC number of the gut could be due to biological mechanisms such as cell growth and death, which are reported to be influenced by the gut microbial status (dysbiosis and symbiosis) and age (Sovran et al., 2019; Gebert et al., 2020). Therefore, the same number of total GC per crypt during the 5 weeks period of this study, could indicate a stable gut environment that does not require production of additional protective mucins (produced by GC). Mucins are the major components of the intestinal mucus layer and can be categorized as acidic or neutral based on their net molecular charge. Acidic type expresses a net negative charge and neutral type exhibits a net neutral charge of the mucin molecule (Derrien et al., 2010). The diverse forms of mucins found in GC may provide clues to host adaptability to gut microbiota (Sicard et al., 2017). In the current study, the majority (61–85 %) of GC was found as a mixed type (containing relatively similar proportion of acidic and neutral mucins) and around 15–39 % of total GC was an acidic type, suggesting that the proportion of secreted acidic mucins in the mucus layer of the caecum may be greater than neutral mucins. Furthermore, the number of acidic GC per crypt increased with age and peaked at 21 days, whereas the amount of mixed GC was unaffected by age. Increasing the overall negative charge (acidic) of mucins enhances mucus viscosity which may be associated with increased gut bacterial diversity and amount of bacterial-derived compounds as age increases. A greater number of GC, particularly acidic GC, could produce more protective mucins that resist bacterial degradation, thus provides more protection against pathogens and mechanical irritation (Montagne et al., 2004). Mucin modifications (including sialylation and sulfation which result in acidic mucins) are typically promoted along with the maturation process of GC (Hino et al., 2012). The observed increase in the proportion of acidic GC and the number of acidic GC per crypt during the first 3 weeks of age may reflect GC maturation, which is important for the functional protection of the intestinal epithelium. This could be attributed to the increased diversity of the caecal microbial community and the increase in feed consumption (more mechanical irritation) of broilers with growing older. It should be noted that individual sampling from tissues of sacrificed birds provides a snapshot of the intestinal response which may cause a bias in either direction for time-sensitive variables such as GC due to their rapid, continuous turnover (3–7 days) at the crypt base (Birchenough et al., 2015). Nevertheless, this study was able to capture the variation of GC proliferation during 14-day periods (from day 7 to day 21, and from day 21 to day 35), particularly for acidic GC. In agreement with prior research, the increased number of GCs and the production of acidic mucins in the small intestine of broilers demonstrated gut maturation of broilers due to exposure to intestinal normal flora over time (Forder et al., 2012).

The mRNA abundance of all cytokines, MUC2 and CLDN5 was also mostly regulated by age, with only a few genes being modified by dietary treatment (IL-10) and breed (IL-1β, IL-6, TNF-α and CLDN5). Throughout the whole study period, the mRNA abundance of all cytokines was increased with age, except IL-1β and TNF-α which reached the highest level at 21 days of age. The alterations in cytokine mRNA abundance in healthy unchallenged broilers may only reflect the interaction between the gut immune system and the commensal microflora (Bar-Shira and Friedman, 2006; Crhanova et al., 2011). The mRNA abundance of several cytokines in the caecum like IL-1β, IL-18, IL-22 and TNF-α was found to be fluctuated due to the colonization of normal gut microflora during first 58 days of age, reflecting an adaptation of the gut immune system to microbiota (Crhanova et al., 2011). In another study, it has been shown that mRNA abundance of IL-1β and TNF-α increased after hatch and decreased during the third week of age (Crhanova et al., 2011), which is in accordance with our study. During the first week after hatching, antigens from the diet and environment constructed an immune response in the caecum of broilers via recruiting granulocyte and T-lymphocyte and generating cytokines, which could trigger immunological adaptation to luminal antigens and microbiota (Van Immerseel et al., 2002; Bar-Shira et al., 2003; Crhanova et al., 2011). Immunological adaptation reduce or eliminate the impacts of dietary and environmental challenges and restore the balance in the immune system (Broom and Kogut, 2018). In the current study, the mRNA abundance of IL-1β and TNF-α as pro-inflammatory cytokines was downregulated after 3 weeks of age, which may imply a lesser degree of immune response in the gut, following transitory inflammatory activation as an immunological adaptation process. It has been also shown in another study that, healthy unchallenged broilers had a lower inflammatory response in the caecal tissue after 3 weeks of age, which was associated with a reduction in potential pathogenic bacteria such as *Escherichia* and *Shigella* and an increase in some beneficial bacteria like *Firmicutes* (including *Faecalibacterium)* in the caecum (Oakley and Kogut, 2016).

Previous studies have shown that bacterial metabolites regulate the immunological pathways of intestinal cells (Yang et al., 2018; Parada Venegas et al., 2019). Therefore, the observed correlations between metabolites and mRNA abundance of cytokines and epithelial barrier proteins in the current study may be interpreted as the relative extent of host-microbiota interactions through bacterial metabolites. Several SCFA mainly propionate and n-butyrate showed an association with cytokines investigated in the caecum (Figure 1). This was in line with in several studies indicating the effects of SCFA on intestinal immunity as reviewed by Gasaly et al. (2021). It was proposed that SCFA may regulate cytokine production of the immune or epithelial cells by directly binding to certain receptors such as free fatty acid receptor (or G protein-coupled receptors) and/or by regulating target cell epigenetics after they were taken up into the cells (Liu et al., 2021). Among SCFA, propionate demonstrated a relatively strong positive correlation with most of cytokines analyzed in this study, especially IL-4, IFN-γ and TGF-β2. The balance between pro-inflammatory (e.g. IFN-γ and IL-4) and anti-inflammatory (e.g. TGF-β) cytokines was shown to regulate mucosal inflammation in response to the presence of bacterial antigens (Neurath et al., 2002). Higher mRNA abundance of TGF-β could be associated with inflammatory inhibition through suppressing cytotoxic actions of Th1 lymphocytes and their cytokines such as IFN-γ, while it simultaneously promoted humoral immune mechanisms of Th2 lymphocytes which stimulate IL-4 secretion (Jarosz et al., 2017). In contrast to SCFA, lactate was negatively correlated with several investigated pro-inflammatory cytokines including IL-1β, IL-4, TNF-α and IFN-γ, thus increasing lactate concentration may inhibit inflammatory response in the caecum of broilers. The anti-inflammatory effects of lactate was also observed in the colon of mice through downregulation of pro-inflammatory cytokines such as IL-1β and TNF-α (Iraporda et al., 2016). Although our result illustrated a potential role of bacterial metabolites on the intestinal immunity, their mechanisms of action are difficult to explain due to their complicated interaction with multiple signaling molecules. Sudden changes in microbial community or activity, such as those caused by stress or pathogenic challenges, can rapidly shift the gut’s immunological balance toward a pro-inflammatory response, inducing a transient physiological inflammation and immune stimulation (Crhanova et al., 2011). The mRNA related to intestinal barrier integrity including MUC2 (mucus protein) and CLDN5 (one of the barrier-forming claudins) also increased their abundance with age in the current study. Several studies suggested that mRNA abundance of MUC2 and CLDN family genes reflect maturation of the intestinal tract during postnatal development (Holmes et al., 2006; Proszkowiec-Weglarz et al., 2020), as well as shifts in bacterial composition in the caecum during animal’s growth (Lu et al., 2003). In the present study, an increased mRNA abundance of MUC2 and CLDN5 occurred along with more developed CD and certain alterations in bacterial metabolites during 35 days of age, which could reflect an increased protective barrier in response to age-related changes in gut microbiota and their metabolic activity. Correlation between bacterial metabolites and CLDN5 was stronger than their correlation with MUC2, with propionate having the strongest correlation with CLDN5. Propionate was also reported to increase the expression of tight junction proteins including ZO-1, CLDN1, CLDN8 and occludin in rat’s colon, hence propionate may enhance intestinal epithelial integrity (Xia et al., 2017).

## 5 Conclusion

Our result showed that bacterial metabolites could play a significant role in mucosal development and immunological response in the caecum of broilers. Bacterial metabolites, particularly SCFA, seemed to contribute to the formation of crypts in the caecum and modify gut immunity over time. The influence of sex on measured variable was completely obscured in this study, while the other genetic-related factor (breed) and dietary treatments (probiotics and phytobiotics) showed a limited impact on microbial metabolites and immunological responses. However, the few observed impacts of breed and dietary treatment as well as interactions between factors on measured variables did not show any meaningful biological pattern. Age remarkably impacted mucosal morphology, goblet cell proliferation and bacterial metabolic activity as well as mRNA abundance of the genes responsible for inflammatory response in the caecum of broilers. This impact could be attributed to the interaction between the gut microbiota and immune system as well as the direct effect of microbial metabolites on the gut histomorphology and cytokine mRNA abundance.

## Data Availability

The original contributions presented in the study are included in the article/Supplementary Material, further inquiries can be directed to the corresponding author. The raw data supporting the conclusions of this article will be made available by the authors, without undue reservation.
