# Supplementary material for: Alterations in bacterial metabolites, cytokines, and mucosal integrity in the caecum of broilers caused by feed additives and host-related factors
Source: Front Physiol. 2022 Sep 12;13:935870. doi: 10.3389/fphys.2022.935870 (PMC9512067; doi:10.3389/fphys.2022.935870)
Supplement: Supplementary file 1 [file DataSheet1.pdf]

Supplementary Material

Table 3a. *p*-values for the effect of interactions between main factors on caecal histomorphology*

Parameters	*p*-value										
	A*T	A*B	A*S	T*B	T*S	B*S	A*T*B	A*T*S	A*B*S	T*B*S	T*B*S*A
Caecal morphology											
CD [1]	0.762	0.427	0.188	0.683	0.639	0.434	0.304	0.611	0.749	0.842	0.412
Goblet cell number [2]											
Acidic	0.193	0.557	0.181	0.119	0.879	0.993	0.928	0.080	0.997	0.764	1.000
Mixed	0.229	0.374	0.838	0.848	0.598	0.498	0.881	0.809	0.975	0.714	0.606
Total	0.124	0.241	0.570	0.742	0.784	0.552	0.869	0.529	0.982	0.869	0.700
Goblet cell density [3]											
Acidic	0.355	0.434	0.304	0.088	0.358	0.597	0.780	0.357	0.824	0.870	0.774
Mixed	0.032	0.147	0.890	0.304	0.162	0.832	0.341	0.398	0.701	0.193	0.038
Total	0.049	0.095	0.940	0.429	0.160	0.680	0.298	0.395	0.648	0.421	0.081

[1] Crypt depth are measured in µm.
[2] the average number of goblet cells per caceal crypt. Acidic represents the cells that are positive to Alcian blue dye. Mixed represents the cells that are positive to both Alcian blue and PAS dye. Total represents the sum of acidic and mixed goblet cells.
[3] The average number of goblet cells per 100 µm length of the crypt depth. Acidic represents the cells that are positive to Alcian blue dye. Mixed represents the cells that are positive to both Alcian blue and PAS dye. Total represents the sum of acidic and mixed goblet cells.
* The main factors consist of three age groups (A; day 7, 21 and 35 of age), three dietary treatments (T; control, probiotic and phytobiotic products), two breeds (B; Ross and Cobb) and two sexes (S; male and female). Data were obtained from samples taken from one bird per pen and subjected to ANOVA using GLM procedure with a 3 × 3 × 2 × 2 (age, dietary treatment, breed and sex) factorial arrangement of experimental groups.

Table 3b. The effect of interaction between age and dietary treatment on the density of mixed and total goblet cells*

Age	Dietary treatment	Mixed GC density [1]	Total GC density [1]
Day 7	CO	9.1 ± 0.77 [ab]	12.9 ± 0.93 [a]
	PO	11.4 ± 1.12 [a]	14.8 ± 1.37 [a]
	PY	9.1 ± 0.99 [ab]	12.5 ± 1.18 [ab]
Day 21	CO	6.4 ± 0.38 [c]	9.5 ± 0.43 [bc]
	PO	6.0 ± 0.33 [c]	9.0 ± 0.45 [c]
	PY	5.6 ± 0.45 [c]	7.9 ± 0.45 [c]
Day 35	CO	5.6 ± 0.43 [c]	7.1 ± 0.53 [c]
	PO	5.6 ± 0.52 [c]	7.3 ± 0.61 [c]
	PY	6.7 ± 0.50 [bc]	8.3 ± 0.52 [c]

[1] The average number of goblet cells per 100 µm length of the crypt depth. Mixed represents the cells that are positive to both Alcian blue and PAS dye. Total represents the sum of acidic (positive to Alcian blue dye) and mixed goblet cells.
[a,b,c] Means with different superscripts in a column differ significantly ($p < 0.05$).
* Results are reported as means of 24 replicate-pens. Treatment means were separated by the Tukey's HSD post hoc test. Three age groups (day 7, 21 and 35 of age) and three dietary treatments (control, probiotic and phytobiotic products) were included in the analysis.
CO = Control, PO = Probiotic product, PY = Phytobiotic product

Table 3c. The effect of interaction between age, dietary treatment, breed and sex on the density of mixed goblet cells*

Age	Dietary treatment	Breed	Sex	Mixed GC density
Day 7	CO	RS	M	8.9 ± 1.82 [abcd]
			F	9.2 ± 1.10 [abcd]
		CB	M	10.8 ± 2.10 [abc]
			F	7.5 ± 0.15 [bcd]
	PO	RS	M	14.8 ± 1.33 [a]
			F	10.9 ± 2.55 [abc]
		CB	M	8.9 ± 1.74 [abcd]
			F	11 ± 2.69 [abc]
	PY	RS	M	8.2 ± 1.13 [bcd]
			F	13.4 ± 2.59 [ab]
		CB	M	8.9 ± 1.74 [abcd]
			F	11 ± 2.69 [abc]
Day 21	CO	RS	M	6.8 ± 0.67 [cd]
			F	6.6 ± 0.52 [cd]
		CB	M	8.9 ± 1.74 [abcd]
			F	11 ± 2.69 [abc]
	PO	RS	M	5.7 ± 0.94 [cd]
			F	5.9 ± 0.48 [cd]
		CB	M	8.9 ± 1.74 [abcd]
			F	11 ± 2.69 [abc]
	PY	RS	M	5.3 ± 1.00 [cd]
			F	5.8 ± 1.22 [cd]
		CB	M	8.9 ± 1.74 [abcd]
			F	11 ± 2.69 [abc]
Day 35	CO	RS	M	5.8 ± 1.01 [cd]
			F	5.4 ± 0.65 [cd]
		CB	M	6.1 ± 0.73 [cd]
			F	5.3 ± 1.13 [cd]
	PO	RS	M	5.7 ± 1.20 [cd]
			F	7.3 ± 1.21 [bcd]
		CB	M	4.4 ± 0.30 [d]
			F	4.7 ± 0.75 [cd]
	PY	RS	M	7.3 ± 0.94 [bcd]
			F	7.2 ± 1.43 [bcd]
		CB	M	4.4 ± 0.30 [d]
			F	4.7 ± 0.75 [cd]

[1] The average number of goblet cells per 100 µm length of the crypt depth. Mixed represents the cells that are positive to both Alcian blue and PAS dye.

[a,b,c,d] Means with different superscripts in a column differ significantly ($p < 0.05$).

* Results are reported as means of 6 replicate-pens. Treatment means were separated by the Tukey's HSD post hoc test. Three age groups (day 7, 21 and 35 of age), three dietary treatments (control, probiotic and phytobiotic products), two breeds (Ross308 and Cobb500) and two sexes (male and female) were included in the analysis.

CO = Control, PO = Probiotic product, PY = Phytobiotic product, RS = Ross, CB = Cobb, M = Male, F = Female

Table 4a. *p*-values for the effect of interactions between main factors on caecal metabolite concentration of broilers*

Parameters	*p*-value										
	**A*T**	**A*B**	**A*S**	**T*B**	**T*S**	**B*S**	**A*T*B**	**A*T*S**	**A*B*S**	**T*B*S**	**T*B*S*A**
Short chain fatty acids											
Acetate	0.525	0.387	0.477	0.466	0.839	0.503	0.694	0.820	0.870	0.955	0.257
Propionate	0.479	0.420	0.242	0.971	0.138	0.685	0.833	0.896	0.745	0.893	0.773
i-butyrate	0.413	0.191	0.229	0.668	0.620	0.477	0.328	0.089	0.669	0.341	0.266
n-butyrate	0.495	0.709	0.750	0.068	0.348	0.318	0.647	0.670	0.952	0.102	0.886
i-valerate	0.850	0.690	0.804	0.649	0.541	0.867	0.303	0.393	0.591	0.831	0.750
n-valerate	0.774	0.556	0.229	0.210	0.159	0.106	0.298	0.602	0.591	0.303	0.334
Total SCFA [1]	0.662	0.375	0.248	0.552	0.758	0.561	0.834	0.938	0.779	0.645	0.411
Total BCFA [2]	0.489	0.241	0.482	0.880	0.486	0.697	0.369	0.098	0.842	0.749	0.425
Lactate											
L -lactate	0.948	0.604	0.249	0.775	0.920	0.768	0.505	0.825	0.672	0.294	0.119
D -lactate	0.860	0.298	0.212	0.892	0.289	0.309	0.450	0.132	0.501	0.088	0.828
Total lactate	0.931	0.825	0.217	0.980	0.508	0.483	0.461	0.326	0.584	0.147	0.789
D - to L -lactate ratio	0.644	0.575	0.563	0.798	0.436	0.386	0.170	0.349	0.656	0.873	0.789

[1] Total short chain fatty acid is the sum of acetate, propionate, i-butyrate, n-butyrate, i-valerate and n-valerate.
[2] Total branched chain fatty acid is the sum of i-butyrate and i-valerate.
* The main factors consist of three age groups (A; day 7, 21 and 35 of age), three dietary treatments (T; control, probiotic and phytobiotic products), two breeds (B; Ross and Cobb) and two sexes (S; male and female). Data were obtained from samples taken from one bird per pen and subjected to ANOVA using GLM procedure with a 3 × 3 × 2 × 2 (age, dietary treatment, breed and sex) factorial arrangement of experimental groups.

Table 5a. *p*-values for the effect of interactions between main factors on mRNA abundance[1] in the caecum of broilers*

Parameters	*p*-value										
	**A*T**	**A*B**	**A*S**	**T*B**	**T*S**	**B*S**	**A*T*B**	**A*T*S**	**A*B*S**	**T*B*S**	**T*B*S*A**
Cytokines											
IL-1β	0.226	0.818	0.096	0.667	0.615	0.791	0.690	0.061	0.756	0.970	0.916
IL-2	0.403	0.660	0.773	0.941	0.952	0.248	0.813	0.977	0.042	0.330	0.445
IL-4	0.940	0.256	0.611	0.787	0.379	0.567	0.215	0.467	0.310	0.977	0.953
IL-6	0.760	0.689	0.883	0.805	0.648	0.476	0.800	0.211	0.962	0.668	0.685
IL-8	0.419	0.976	0.626	0.329	0.569	0.565	0.976	0.921	0.891	0.504	0.897
IL-10	0.371	0.996	0.178	0.295	0.625	0.369	0.281	0.992	0.776	0.595	0.301
IL-12	0.477	0.974	0.292	0.531	0.260	0.797	0.483	0.795	0.449	0.942	0.208
IL-17α	0.710	0.683	0.911	0.586	0.409	0.430	0.527	0.413	0.163	0.112	0.065
IL-18	0.467	0.736	0.927	0.397	0.498	0.412	0.517	0.309	0.630	0.130	0.715
TNF-α	0.238	0.848	0.914	0.175	0.050	0.422	0.306	0.557	0.471	0.173	0.783
IFN-γ	0.366	0.743	0.572	0.540	0.631	0.845	0.873	0.635	0.988	0.166	0.434
TGF-β2	0.839	0.503	0.514	0.434	0.203	0.162	0.703	0.345	0.136	0.448	0.976
Gut barrier related proteins											
MUC2	0.274	0.842	0.137	0.241	0.577	0.145	0.057	0.049	0.815	0.766	0.411
CLDN5	0.185	0.531	0.865	0.237	0.128	0.514	0.748	0.220	0.252	0.605	0.819

[1]IL = interleukin, TNF-α = tumor necrosis factor alpha, IFN-γ = interferon gamma, TGF- β = transforming growth factor beta, CLDN5 = Claudin 5 and MUC2 = Mucin 2
* The main factors consist of three age groups (A; day 7, 21 and 35 of age), three dietary treatments (T; control, probiotic and phytobiotic products), two breeds (B; Ross and Cobb) and two sexes (S; male and female). Data were obtained from samples taken from one bird per pen and subjected to ANOVA using GLM procedure with a 3 × 3 × 2 × 2 (age, dietary treatment, breed and sex) factorial arrangement of experimental groups.

Table 5b. The effect of interaction between age, dietary treatment and sex on mRNA expression of MUC2 (log_{10} copy number per ng of RNA) in the caecum of broilers*

Age	Dietary treatment	Sex	MUC2
Day 7	CO	M	-0.90 ± 0.071 [abc]
		F	-0.83 ± 0.115 [ab]
	PO	M	-0.78 ± 0.122 [a]
		F	-0.87 ± 0.109 [abc]
	PY	M	-0.82 ± 0.065 [ab]
		F	-0.80 ± 0.100 [a]
Day 21	CO	M	-1.04 ± 0.115 [abc]
		F	-1.51 ± 0.088 [d]
	PO	M	-1.05 ± 0.087 [abc]
		F	-1.11 ± 0.112 [abcd]
	PY	M	-1.32 ± 0.059 [cd]
		F	-1.26 ± 0.097 [bcd]
Day 35	CO	M	-0.79 ± 0.067 [a]
		F	-0.65 ± 0.078 [a]
	PO	M	-0.71 ± 0.074 [a]
		F	-0.71 ± 0.068 [a]
	PY	M	-0.66 ± 0.065 [a]
		F	-0.66 ± 0.096 [a]

[a,b,c,d] Means with different superscripts in a column differ significantly ($p < 0.05$).
* Results are reported as means of 12 replicate-pens. Treatment means were separated by the Tukey's HSD post hoc test. Three age groups (day 7, 21 and 35 of age), three dietary treatments (control, probiotic and phytobiotic products) and two sexes (male and female) were included in the analysis.
CO = Control, PO = Probiotic product, PY = Phytobiotic product, M = Male, F = Female

Table 5c. The effect of interaction between age, breed and sex on mRNA abundance of IL-2 (log_{10} copy number per ng of RNA) in the caecum of broilers*

Age	Breed	Sex	IL-2
Day 7	RS	M	-5.02 ± 0.046^{bc}
		F	-4.98 ± 0.054^{b}
	CB	M	-4.97 ± 0.064^{b}
		F	-5.03 ± 0.052^{bcd}
Day 21	RS	M	-4.94 ± 0.056^{b}
		F	-5.18 ± 0.075^{cd}
	CB	M	-5.21 ± 0.141^{d}
		F	-5.08 ± 0.068^{bcd}
Day 35	RS	M	-4.01 ± 0.035^{a}
		F	-4.08 ± 0.047^{a}
	CB	M	-4.04 ± 0.053^{a}
		F	-4.11 ± 0.045^{a}

[a,b,c,d] Means with different superscripts in a column differ significantly ($p < 0.05$).
* Results are reported as means of 18 replicate-pens. Treatment means were separated by the Least Significant Difference test. Three age groups (day 7, 21 and 35 of age), two breeds (Ross and Cobb) and two sexes (male and female) were included in the analysis.
RS = Ross, CB = Cobb, M = Male, F = Female

Table 6. The correlation analysis between bacterial metabolites and mRNA abundance[1] in the caecum of broilers*

Correlation coefficients	IL-1β	IL-2	IL-4	IL-6	IL-8	IL-10	IL-12	IL-17α	IL-18	TNF-α	IFN-γ	TGF-β2	MUC2	CLDN5
Acetate	0.027	0.069	0.185	-0.101	0.083	0.088	0.099	0.125	0.121	-0.065	0.191	0.157	-0.024	0.077
Propionate	0.212	0.410	0.409	0.150	0.197	0.414	0.467	0.245	0.518	-0.245	0.246	0.548	0.037	0.449
i-butyrate	0.010	0.111	-0.001	0.071	0.096	0.130	0.145	0.081	0.181	-0.197	0.106	0.127	-0.050	0.151
n-butyrate	0.038	0.129	0.236	0.016	0.120	0.227	0.162	0.151	0.188	-0.130	0.220	0.191	-0.008	0.178
i-valerate	-0.116	0.234	-0.087	0.173	0.148	0.235	0.235	0.197	0.244	-0.285	0.145	0.129	0.163	0.252
n-valerate	0.215	0.294	0.357	0.100	0.208	0.366	0.412	0.215	0.454	-0.154	0.348	0.434	0.026	0.387
Total SCFA[2]	0.058	0.181	0.264	-0.027	0.146	0.232	0.235	0.200	0.243	-0.159	0.249	0.283	-0.003	0.212
Total BCFA[3]	-0.053	0.187	-0.032	0.129	0.126	0.196	0.203	0.145	0.225	-0.266	0.123	0.143	0.044	0.216
L-lactate	-0.189	0.166	-0.015	0.032	0.089	0.103	0.136	0.118	0.007	-0.190	-0.080	0.140	0.167	0.183
D-lactate	-0.324	0.073	-0.179	0.106	0.033	0.020	-0.020	0.012	-0.130	-0.209	-0.227	-0.023	0.126	0.072
Total lactate	-0.279	0.106	-0.175	0.087	0.083	0.061	0.032	0.089	-0.094	-0.198	-0.170	0.003	0.166	0.095
D- to L-lactate ratio	-0.264	-0.054	-0.159	-0.115	-0.098	-0.057	-0.183	-0.153	-0.178	-0.139	-0.232	-0.091	0.008	-0.062
***p*-value**	IL-1β	IL-2	IL-4	IL-6	IL-8	IL-10	IL-12	IL-17α	IL-18	TNF-α	IFN-γ	TGF-β2	MUC2	CLDN5
Acetate	0.710	0.346	0.011	0.169	0.256	0.233	0.174	0.089	0.098	0.371	0.009	0.031	0.748	0.292
Propionate	0.003	<0.001	<0.001	0.041	0.007	<0.001	<0.001	<0.001	<0.001	<0.001	<0.001	<0.001	0.617	<0.001
i-butyrate	0.887	0.128	0.984	0.333	0.189	0.077	0.047	0.270	0.013	0.007	0.148	0.083	0.500	0.038
n-butyrate	0.604	0.079	0.001	0.830	0.099	0.002	0.026	0.038	0.010	0.075	0.002	0.009	0.914	0.014
i-valerate	0.113	<0.001	0.236	0.018	0.042	<0.001	<0.001	0.007	<0.001	0.001	0.047	0.079	0.028	<0.001
n-valerate	0.003	<0.001	<0.001	0.170	0.004	<0.001	<0.001	0.003	<0.001	0.034	<0.001	<0.001	0.730	<0.001
Total SCFA	0.429	0.013	<0.001	0.716	0.045	<0.001	<0.001	0.006	<0.001	0.029	<0.001	<0.001	0.971	0.003
Total BCFA	0.468	0.010	0.663	0.078	0.085	0.007	0.005	0.047	0.002	<0.001	0.091	0.049	0.557	0.003
L-lactate	0.010	0.025	0.843	0.666	0.231	0.170	0.068	0.112	0.931	0.010	0.284	0.060	0.026	0.013
D-lactate	<0.001	0.327	0.015	0.152	0.656	0.787	0.790	0.874	0.078	0.004	0.002	0.758	0.094	0.333
Total lactate	<0.001	0.153	0.018	0.242	0.261	0.417	0.665	0.228	0.201	0.007	0.021	0.971	0.026	0.199
D- to L-lactate ratio	0.003	0.543	0.074	0.198	0.271	0.528	0.038	0.086	0.045	0.116	0.009	0.309	0.926	0.487

[1] IL = interleukin, TNF-α = tumor necrosis factor alpha, IFN-γ = interferon gamma, TGF- β = Transforming growth factor beta, CLDN5 = Claudin 5 and MUC2 = Mucin 2

[2] Total short chain fatty acid is the sum of acetate, propionate, i-butyrate, n-butyrate, i-valerate and n-valerate concentration.

[3] Total branched chain fatty acid is the sum of i-butyrate and i-valerate concentration.

* Data were obtained from one broiler chicken per pen (72 pens) at three different ages (day 7, 21 and 35 of age). Birds in these pens were from two commercial breeds (Ross and Cobb) and two sexes (male and female) and received three dietary treatments (control, probiotic and phytobiotic products). All data were analyzed by Spearman's rank correlation analysis.
